# Dysregulated Expression of Long Intergenic Non-coding RNAs (LincRNAs) in Urothelial Bladder Carcinoma

**DOI:** 10.22088/BUMS.6.4.212

**Published:** 2017-11-12

**Authors:** Zahra Ousati Ashtiani, Gholamreza Pourmand, Seyed Alireza Salami, Mohsen Ayati, Javad Tavakkoly-Bazzaz

**Affiliations:** 1 *Urology Research Center, Tehran University of Medical Sciences, Tehran, Iran.*; 2 *Department of Medical Genetics, School of Medicine, Tehran University of Medical Sciences, Tehran, Iran.*; 3 *Department of Biotechnology, University of Tehran, Tehran, Iran.*; 4 *Uro-Oncology Research Center, Tehran University of Medical Sciences, Tehran, Iran.*

**Keywords:** Bladder cancer, long intergenic non- coding RNA (lincRNA), *LINC00152*, *LINC01082*

## Abstract

Long intergenic non-coding RNA (lincRNA) has been introduced as key regulators of diverse biological processes, including transcription, chromatin organization, cell growth and tumorigenesis. With regard to the potential role of lincRNAs in cancer development, one may postulate that differential expression of lincRNAs could be employed as a tool in cancer diagnosis, prognosis, and targeted therapy. In this study, we aimed to explore the putative correlation between the expression levels of two lincRNAs: *LINC00152* and *LINC01082* in the bladder cancer (BC), in comparison with its adjacent non-cancerous tissue. Fifty Iranian subjects diagnosed with BC, representing in different stages and grades participated in this study The mRNA expression levels of the abovementioned lincRNAs were comparatively analyzed in cancerous and their adjacent non-cancerous counterpart tissues, of each subject by Real-Time PCR. The expression levels of *LINC00152*, and *LINC01082* were significantly lower in tumor tissues in comparison with their adjacent normal tissues (P<0.001). More notably, in the case of *LINC01082* the reduced expression was differentiated by the muscle invasiveness pattern of the tumor (P= 0.05). Our study presents a new finding about the tumor suppressor potentiality of these lincRNAs in BC development that in turn may suggest them as candidate biomarkers. Replicating this study in higher number of BC subjects, coupled with functional analysis, is necessary to investigate interconnections between these RNAs and cancer development, leading to better understanding of cancer biology.

Urothelial bladder carcinoma (BC) is one of the most common cancers of urinary system in the world (1). There are two major types of BC: non-muscle- invasive (NMIBC) or superficial bladder tumors which accounts for approximately 75% of neoplasms, and muscle invasive (MIBC) (2, 3). Appreciation of the molecular mechanisms that are engaged in both BC initiation and development, and also introducing potential markers for early diagnosis and prognosis, is very crucial in patient care.

Recent investigations using high throughput technologies such as whole-genome and RNA sequencing have identified a comprehensive prospect of molecular signatures (4). Large-scale sequencing revealed a great number of noncoding RNAs, comprising small/short and long noncoding RNAs. It is estimated that nearly 98% of transcribed regions composed of noncoding RNAs. They contribute to a variety of biological functions, protect genomes from foreign nucleic acids, and can conduct DNA synthesis or genome rearrangement (5). Long non-coding RNAs (lncRNAs) is a newly-identified class of RNAs with 0.2 to 100 kb size, often capped and polyadenylated, and lacking open reading frame. They can be nuclear or cytoplasmic or both, and participate in gene regulation at the transcriptional or post-transcriptional levels, and are also actively involved in cell proliferation, differentiation, and apoptosis (6-8). Recently it has been demonstrated that lncRNAs interact with chromatin at more than thousands diver locations across multiple chromosomes, and modulate large-scale gene expression programs (9). LncRNAs biology has attracted a great attention in cancer genome research. There are evidences showing their roles in the pathophysiology of diseases, and also in the development and progression of human cancers. They have the potential to be used as a marker for early diagnosis, and molecular targeted therapy. Thousands of lncRNAs have been identified (10, 11). A microarray analysis of the lncRNAs expression profile in BC detected 1,122 differentially expressed lncRNAs; among these, 734 lncRNAs were upregulated and 388 were downregulated (12). Genetically, lncRNAs are classified into sense, antisense, bidirectional, intronic, and intergenic. There are some reports that indicate lncRNAs play an oncogenic role in BC (10-14). Long intergenic noncoding RNAs (LincRNAs) are transcribed from non-coding DNA sequences between coding genes and do not overlap exons and other transcripts. LincRNAs that are localized in nucleus are involved in regulating gene transcription, and chromatin organization (9). In primary tumors and metastases, they show distinct gene expression patterns. Also, lincRNAs may affect human diseases and epigenetic information, the latter may affect cellular growth (15). There are over 5,000 lincRNAs that were annotated across human tissues using high-throughput sequencing technologies (16), but a small portion of them have been characterized. Despite the fact that lincRNAs are being considered as key regulators of different cellular processes, molecular mechanisms of action and the function of individual lincRNAs stay a challenge. It is reported that differences in expression of lincRNAs between normal and cancer cells are associated with cancer progression (15). Therefore, differential expression of lincRNAs can be useful for cancer diagnosis, prognosis and targeted therapy. *LINC01082* is thought to be involved in long-range control of chromatin structure, and gene expression (17). It is reported that *LINC00152* which is a new lincRNA, may participate in cell cycle arrest, apoptosis, epithelial to mesenchymal transition, cell migration, and may be used as a reliable biomarker for some cancer types diagnosis (18, 19). Relatively, in colon cancer tissues, *LINC 00152* increased expression is associated with poor prognosis, and in gastric cancer is correlated with invasion, lymph node metastasis and poor survival (20, 21). Decreased expression of *LINC 00152* has been observed in colorectal cancer (CRC) tissue and CRC cell line (22).

Regarding lncRNA features such as tissue-specific expression and dysregulation of lncRNAs in a variety of cancers especially their misexpression in solid tumor (15), we aimed to examine the differential expression of two lincRNAs; *LINC00152 *and *LINC01082* in BC tumors and their adjacent normal tissue samples taken from the same subject.

## Materials and methods

**Subjects and tissue samples**

After being informed with the aim and the methods used in this study, each patient participating in this project signed a written informed consent form. Paired bladder tumor and adjacent normal tissue samples were obtained from 50 Iranian individuals who underwent transurethral resection of bladder tumor or radical cystectomy at the Sina and Imam Khomeini Hospitals. All samples were pathologically evaluated according to the TNM and World Health Organization classification by two experienced pathologists. Cancerous and their adjacent non-cancerous samples as normal control from the bladder were quickly frozen in liquid nitrogen following collection, and were stored at -80^o^C until subsequent RNA extraction.

Of the 50 patients, 43 were males and 7 were females. The mean age was 67.1 ± 8 years. None of the patients received any antitumoral treatment (BCG therapy, radiotherapy or chemotherapy) prior to surgery and sample collection. Clinicop-athological information such as grade, stage, age, gender, smoking, family cancer history, was provided for all subjects. This study was approved by Research Review Board and also the Ethics Committee of Tehran University of Medical

Sciences (TUMS).

**RNA Extraction and cDNA synthesis**

Total RNA from tumor and adjacent non-tumor tissues were extracted by TriPure Isolation Reagent (Roche Life Science, Germany) according to manufacturer's instructions. Concentration and purity of the total RNA were estimated spectrophotometrically by measuring its optical density (A260/280>2.0; A260/230>1.8) using NanoDrop-2000 (Thermo, USA), and agarose gel electrophoresis. Possible DNA contamination within the samples was *removed by* DNase I (Thermo Fisher Scientific, USA) treatment.

One μg of total RNA was reversely transcribed to cDNA using PrimeScript^TM ^RT reagent kit (Takara, Japan). Thermal Cycler (SensoQuest GmbH, Germany) was used for incubating the reaction mixture at 37 ^o^C for 15 min and 85^ o^C for 5 s. Prepared cDNAs were stored at -20 C until further use. All steps were performed according to the manufacturer’s recommendation.

**Gene expression analysis**

Specific sets of primers were designed for *LINC00152*, and *LINC01082* for gene expression analysis. Housekeeping Glyceraldehyde 3-phosphate dehydrogenase (*GAPDH*) gene was used for normalization. All amplicons length for real-time PCR were less than 200 base pairs. Primer sets were checked using primer-BLAST and Oligo analyzer software. The primers sequence and amplicon length are presented in Table 1.

**Table 1:** List of primer sets for Real-Time PCR

**Primers Sequence (5´ 3´) Amplicon size (bp)**		
LINC00152-F	AGACACCGAAAATCACGACTC A	146
LINC00152-R	AGACCACCCGCAAATGCAGA	
LINC01082-F	CAGTGACAG CCTTAACATTTG C	141
LINC01082-R	TTCGGTGCTGGGTTGATCCT	
GAPDH- F	ATC CTG GGCTACACTGAG C	159
GAPDH- R	CACCACCCT GTT GCTGTA G	

Real-Time PCR was performed in a total volume of 20 μl using HiFi SYBR Green Master Mix (Thermo Fisher Scientific, USA). The following thermal cycling conditions were used: incubation at 95 °C for 10 min followed by 40 cycles of 95 C for 15 s, 60 C for 20 s, and 72 C for 20 s. Amplification reactions were performed in triplicate for all samples. The average value in each triplicate was used to calculate the relative amount of expression. A melting curve was obtained following amplification. No template control (NTC) (nuclease-free water) was included in each run. Quantitative PCR (qPCR) analysis was completed using Rotor-Gene^TM ^6000 (Corbett Life Science, Australia). Ct values were collected for the genes of interest and *GAPDH* as housekeeping gene during the log phase of the cycle. Following amplification reaction, the amplification and melting curves were analyzed. To determine the specificity of the Real-Time PCR reaction products, agarose gel electrophoresis was applied. Efficiencies of reference and target genes were nearly equal. Gene expression data analysis was carried out using the comparative threshold cycle number (2^-ΔΔCT^) method according to the following formula: ∆ct1= ct target– ct housekeeping, ∆ct2= ct normal– ct housekeeping, ∆∆ct=∆ct1-∆ct2

**Statistical analysis**

Statistical analysis was performed using SPSS software version 21. Kolmogorov-Smirnov test was performed to assess the normality of quantitative data. The expression level of two lincRNAs in tumor and normal tissues, and their association were analyzed by non-parametric Mann-Whitney, and Spearman tests. Multivariate linear regression analysis was performed to find out any significant relation between clinicopathological parameters, and relative expression. In this regression all clinicopathological variables including independent variables were assessed using stepwise method. A P-value was set at 0.05 or less to indicate statistically significant difference.

## Results

A total of 50 BC patients including 43 (86%) men and 7(14 %) women participated in this study. Their median age was 67 years (range 49-85 years). Among the patients, 72% had high-grade, and 28% had low-grade tumors with different stages. There were 34 (68%) patients with the smoking habit, and were all cigarette smokers for more than 10 years. 20% (10/50) of the cases were opium addict, 24% (12/50) had diabetes, and 56% (28/50) showed cardiovascular and/or respiratory diseases. The rate of occupational exposure was about 54% (27/50). The demographic and clinicopathological parameters of all patients are summarized in Table 2.

**Expression analysis of lncRNAs**

**Fig. 1:**
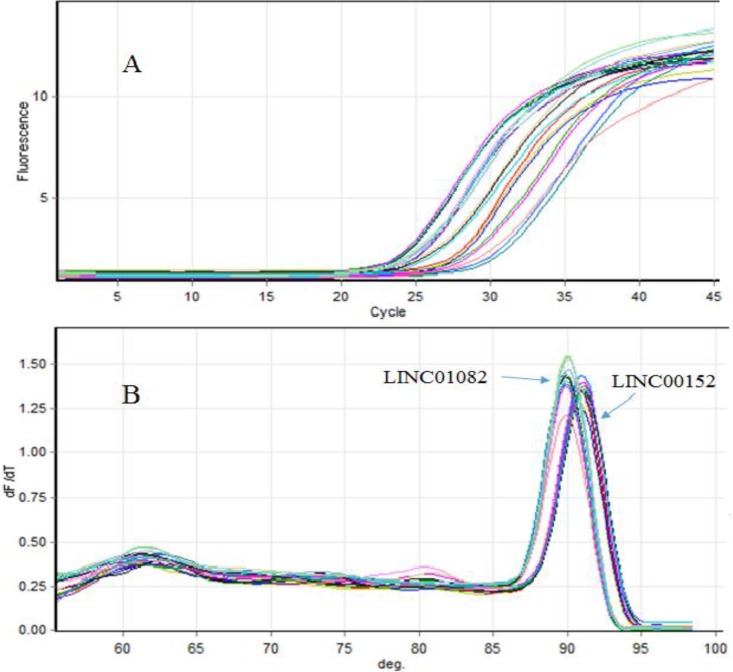
Real-Time PCR assay for lincRNAs. A: relative fluorescence vs cycle number; B: melt curves from qPCR of LINC00152, and LINC01082

The relative gene expression level of *LINC00152* and *LINC01082* were compared between the bladder cancerous and their nearby non-cancerous tissues by real-time PCR. Melting curves of two lncRNAs are demonstrated in Figure1.

**Fig. 2:**
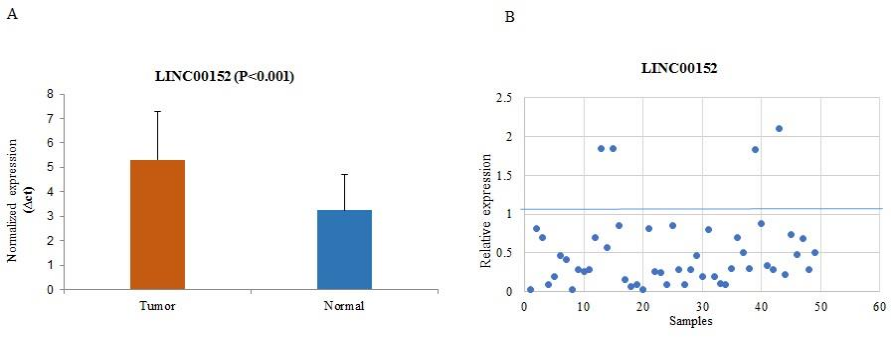
Normalized expression of LINC00152 in cancerous and normal tissues of BC. A: the higher ∆Ct shows lower expression; B: relative expression of LINC00152 in each tumor tissue compared with non-cancerous tissue

*LINC00152* expression level was downregulated among 92% (46/50) of cases regardless the stage and grade of the tumor. Figure2* demonstrates normalized expression of LINC00152 in tumor and normal tissues (the higher ∆ct shows lower expression), and relative expression of each sample.* Statistical non-parametric test (Mann-Whitney) confirmed that the expression levels of *LINC00152* in tumoral and non-tumoral tissues had significant difference (fold change:-2.2; P<0.001). The association between decreased expression and clinicopathological parameters in 50 samples did not touch statistical significance except for occupation exposure (P=0.028).

**Fig. 3:**
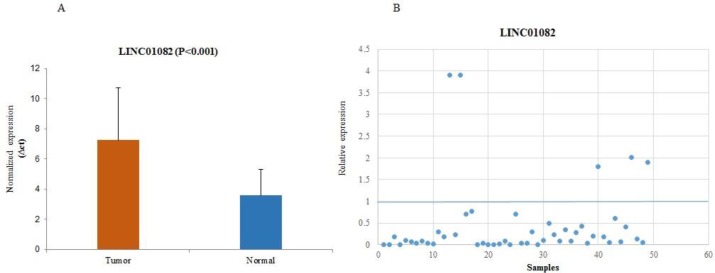
Normalized expression of LINC01082 in cancerous and normal tissues of BC. A: the higher ∆Ct shows lower expression; B: relative expression of LINC01082 in each tumor tissue compared with non-cancerous tissue

**Fig. 4:**
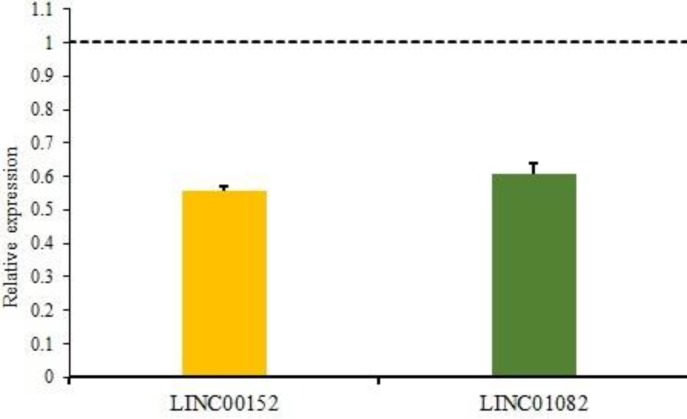
Relative expression of LINC00152 and LINC01082. Both lincRNAs were downregulated in BC

Comparison of *LINC01082* expression level in cancerous and adjacent normal tissues also showed significant difference (fold change:- 2.2; P<0.001) and was downregulated in 90% (45/50) of cases. Statistical analysis demonstrated that there were relations between decreased expression of* LINC01082* with tumor type (P= 0.05), and tumor grade (P=0.009). Normalized expression of *LINC01082* in tumoral and non-tumoral tissues are shown in Figure 3. Relative expression of both lincRNAs are shown in Figure 4.

**Table 2:** Clinicopathological characteristics of the patients

Clinicopathological parameters	Number	percent
Age (year)		
≤67	26	52
>67	24	48
Sex		
Male	43	86
Female	7	14
Stage		
I	16	32
II	20	40
III	10	20
IV	4	8
Grade		
Low	14	28
High	36	72
Tumor type		
Non-invasive muscle	15	30
Muscle invasive	35	70
Family history		
Bladder cancer	8	16
Other cancer	6	12
No cancer	36	72
Smoker		
yes	34	68
No	16	32
Occupational exposure	27	54

**Table 3:** Differential expression of LncRNAs in bladder cancer

**LncRNA type**	**LncRNA**	**Chr:Start-End** **Genomic coordinates (GRCh38)**	**Expression**	**P-value**
Intergenic	*LINC00152*	2: 87455455-87521518	Down	<0.001
Intergenic	*LINC01082*	16: 86196180-86199719	Down	<0.001

Analysis by multivariable regression method with stepwise variable selection verified the association of *LINC00152* expression with age (P= 0.022), and occupation exposure (P= 0.002), and *LINC01082* expression with tumor grade (P= 0.009). Table 3 summarizes the location and expression of these lincRNAs with P-values from Mann-Whitney test.

Spearman correlation test among expression levels of *LINC00152*, and *LINC01082* showed that there was a significant correlation between *LINC00152*, and *LINC01082* (P= 0.032).

## Discussion

This is the first study that addressed *LINC00152* and *LINC01082* expression levels in Iranian BC patients. We described dysregulation of these lincRNAs expression in tumoral tissues in comparison with non-tumoral tissues. Our results represent a correlation between some of clinicopathological parameters and these lncRNAs. Although lincRNAs tend to have low expression levels, and have tissue specific or cell specific feature, but they are involved in cancer pathogenesis as oncogene or tumor suppressor and it is suggested that they can act as key regulators of diverse biological processes (23, 24). They could govern cellular functions in different occasions from embryonic stem cell division to progression of cancer (24, 25).

In the present study, the expression level of *LINC00152* in BC tissues in comparison with their matched adjacent non-tumoral tissues were decreased im most cases (P<0.001). Our results are in accordance with Zhang et al.’s report on colorectal cancer, where *LINC00152* expression significantly decreased in CRC tissues (examined in 49 subjects and also CRC cell lines), and this change was more frequently observed in patients with advanced stages of the disease. Whereas, there are reports on gastric cancer and tongue squamous cell carcinoma that demonstrated upregulation of this lincRNA. Although upregulation of *LINC00152* has been observed in lung adenocarcinoma, but in half of the patients (30/60), its level decreased (26). Eight percent of our patients showed also upregulation. In gastric cancer, increased expression positively correlated with larger tumor size (18, 27). The difference may be due to the fact that the majority of lincRNAs are expressed in a highly cell/tissue-specific manner, and therefore may realize their paradoxical oncogenic or tumor suppressor influences. Such discrepancies could be partly attributed to epigenetic changes, and also histopathological heterogeneity within individual tumor type, grade, and stage. Based on the increased expression of *LINC00152* in some cancers such as gastric cancer, hepatocellular carcinoma, colon cancer, and gallbladder cancer, an oncogenic role for this lincRNA has been contemplated (28). Regarding our observation, compatible with another study conducted on CRC tissues, and CRC cell line, we suggest that *LINC00152* may play this time a distinctive role as a tumor suppressor. In our study, downregulation of *LINC00152* was associated with occupation exposure (P= 0.028), and age (P= 0.022). It is reported that occupational exposure to contaminants can lead to epigenetic changes which in turn affect transcription potential, and may result in cancer (29, 30). Direct or indirect dysregulation of lincRNAs can cause epigenetic changes (31) that offer insights into the special value of epigenetic mechanisms in BC. In other word, there is association between lncRNA dysregulation and epigenetic alterations in cancer. Since molecular mechanisms, and the function of individual lincRNAs stay a challenging task, and the biological roles of *LINC00152* are mainly unknown in BC pathogenesis, further functional studies are needed to identify the exact role of *LINC00152* in BC.

Downregulation of *LINC01082* expression was also observed (P<0.001) in 90% of cases that was associated with tumor type (P= 0.05). LincRNAs such as *LINC01082*, are thought to be involved in long-range control of chromatin structure, and gene expression. Some of the lincRNAs play role as transcriptional regulators, and can act locally to regulate the expression of neighboring genes, or the genes in far distance across multiple chromosomes (32, 33). *LINC01082* gene is located upstream and close to *FOXF1 *gene which encodes a transcription factor. Therefore, we suppose that *LINC01082* may act as a transcriptional regulator. In addition, deletion in *FOXF1* gene and removing part of this lincRNA lead to *FOXF1* expression decrease in lung disease, which suggests that it may be a distant regulator (17, 34, 35). Since statistical analysis demonstrated that there is a correlation between *LINC00152* and *LINC01082* expression (P= 0.032), we hypothesize that these two lincRNAs may act in a similar way in BC.

Our findings represent dysregulation of new lincRNAs in BC, and confirm that they may play a decisive role in cellular processes and tumorigenesis. As valuable tools, lincRNAs may attain new applications for BC clinical management. Moreover, association of lincRNA and occupation exposure with regard to the fact that dysregulation of lincRNA may induce epigenetic changes, could propose the role of epigenetic mechanisms in BC development. Finally, further functional study is recommended to elaborate the exact and distinctive roles of *LINC00152* and *LINC01082 *that could pave the way for better understanding of cancer biology in terms of its development and treatment. 

## Conflict of interest

The authors declare that there is no conflict of interest in this study.

This research was supported by a grant from Tehran University of Medical Sciences (TUMS) (Grant No. 26562).
